# Understanding Sulfur Redox Mechanisms in Different Electrolytes for Room-Temperature Na–S Batteries

**DOI:** 10.1007/s40820-021-00648-w

**Published:** 2021-05-04

**Authors:** Hanwen Liu, Wei-Hong Lai, Qiuran Yang, Yaojie Lei, Can Wu, Nana Wang, Yun-Xiao Wang, Shu-Lei Chou, Hua Kun Liu, Shi Xue Dou

**Affiliations:** Institute for Superconducting and Electronic Materials, Australian Institute of Innovative Materials, University of Wollongong, Innovation Campus, Squires Way, North Wollongong, NSW 2500 Australia

**Keywords:** Room-temperature sodium–sulfur batteries, Carbonate ester electrolyte, Ether electrolyte, Sulfur cathode, Sulfur redox reactions

## Abstract

This work reports influence of two different electrolytes, carbonate ester and ether electrolytes, on the sulfur redox reactions in room-temperature Na–S batteries. Two sulfur cathodes with different S loading ratio and status are investigated. A sulfur-rich composite with most sulfur dispersed on the surface of a carbon host can realize a high loading ratio (72% S). In contrast, a confined sulfur sample can encapsulate S into the pores of the carbon host with a low loading ratio (44% S). In carbonate ester electrolyte, only the sulfur trapped in porous structures is active via ‘solid–solid’ behavior during cycling. The S cathode with high surface sulfur shows poor reversible capacity because of the severe side reactions between the surface polysulfides and the carbonate ester solvents. To improve the capacity of the sulfur-rich cathode, ether electrolyte with NaNO_3_ additive is explored to realize a ‘solid–liquid’ sulfur redox process and confine the shuttle effect of the dissolved polysulfides. As a result, the sulfur-rich cathode achieved high reversible capacity (483 mAh g^−1^), corresponding to a specific energy of 362 Wh kg^−1^ after 200 cycles, shedding light on the use of ether electrolyte for high-loading sulfur cathode.

**Supplementary Information:**

The online version contains supplementary material available at 10.1007/s40820-021-00648-w.

## Highlights

- A ‘solid–liquid’ conversion for increasing the sulfur content from ~ 50 to 72% for RT Na–S batteries is developed.
- The redox mechanisms of two types of sulfur: sulfur on the surface of a cathode host (155S) and sulfur in the pores of the host (300S) in ether and carbonate ester electrolytes are studied.
- The function of NaNO_3_ additive on modifying Na anode and confining the shuttle effect of dissolving polysulfides during ‘solid–liquid’ conversion is visualized.

**Supplementary Information:**

The online version contains supplementary material available at 10.1007/s40820-021-00648-w.

## Graphical abstract

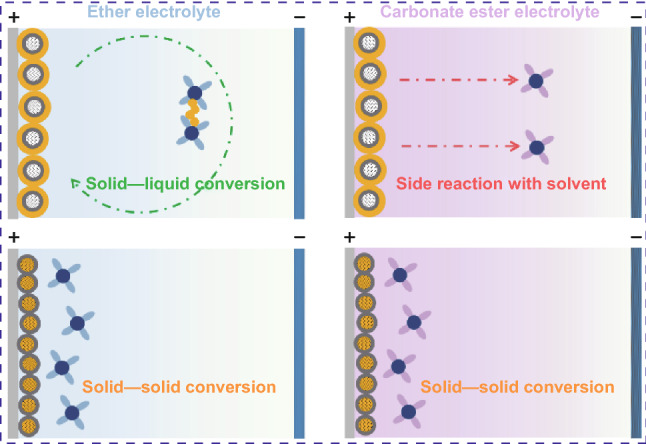

## Introduction

The room-temperature sodium–sulfur (RT Na–S) batteries as emerging energy system are arousing tremendous interest [1–7]. Compared to other energy devices, RT Na–S batteries are featured with high theoretical energy density (1274 Wh kg^−1^) and the abundance of sulfur and sodium resources [8–16]. However, two main problems are important for the development of the RT Na–S batteries in comparison with Li–S batteries [17, 18]. The first one is the S cathode: In recent studies on RT Na–S batteries, the sulfur tended to be implanted into a specific carbon host via thermal treatment (above 155 °C). The resulting sulfur content was mainly stored in the pores of the cathode host with a small amount of sulfur dispersed on the surface [19–21]. To improve the cyclability, catalysts with strong adsorption were also doped into the host to confine the shuttle effect of dissolved polysulfides (PSs) and improve cyclability [6]. This method effectively improves the sluggish kinetics and poor conductivity of sulfur, but the problem is that the resulting sulfur content is low, accounting for around 50% of the total mass. Meanwhile, the catalysts further increase the mass of the electrode, causing low energy density. To address the low sulfur content in the cathode, recent studies on the Li–S batteries usually sulfurated the cathode host at the relatively low temperature of 155 °C for 12 h, and the resulting sulfur content was usually above 70% [22, 23]. The high sulfur content is beneficial to the actual energy density, which is one of the most important parameters for practical applications [24]. The second problem is the electrolyte: Most of studies on RT Na–S batteries usually apply a carbonate ester electrolyte, ethylene carbonate/diethyl carbonate/propylene carbonate (EC/DEC/PC) with the addition of fluoroethylene carbonate (FEC) additive, which is helpful for forming a stably solid–electrolyte interphase (SEI) and obtaining stable cyclability, although the side reactions between sodium polysulfides (NaPSs) and carbonate ester electrolyte cause low initial Coulombic efficiency (CE) [25]. Meanwhile, the high price of carbonate ester electrolyte also reduces commercial interest in the RT Na–S batteries [26]. For Li–S batteries, the S cathode employed ‘solid–solid’ conversion with S confined in microporous carbon host while performed ‘solid–liquid’ conversion with S stored in mesoporous carbon host [27]. The ‘solid–solid’ conversion usually took place in molecular sulfur (S_2–4_), atomic sulfur (sulfurized polyacrylonitrile (SPAN) as a representative) or in electrolytes that were non- or sparingly solvated towards polysulfides (PSs) with conventional C/S composite cathodes [28–32]. In this case, however, the slow kinetics, low sulfur content, and side reactions hinder its further applications [33]. Recent studies found that an ether electrolyte, 1,3-dioxolane/1,2-dimethoxyethane (DOL/DME = 1:1), was more promising in application with a dual discharging platform [34–36]. DME offers high lithium polysulfides (LiPSs) solubility and fast reaction kinetics, while DOL forms a more stable solid–electrolyte interface on the Li surface and provides low PS solubility [37]. With the addition of LiPF_6_ salt and LiNO_3_ additive, a uniform SEI is formed on the surface of the Li metal, which allows dissolved PSs undergo reversible reactions during cycling, resulting in a dual voltage platform (around 2.3 and 2.1 V) in the discharging process with high sulfur content (above 70%) [33, 38, 39]. Learning from Li–S batteries, the S content in cathode should be over 70% to achieve practical expectations. In such a high S content, partial S has to disperse on the surface of cathode host, which inevitably causes side reaction between polysulfides and carbonate ester solvent [27]. The side reaction not only consumes electrolyte but significantly reduces reversible capacity. Therefore, it is necessary to develop different electrolytes and study the S redox mechanism in RT Na–S batteries.

In this work, we increase the sulfur utilization from ~ 44 to 72% for the RT Na–S batteries via ‘solid–liquid’ conversion in ether electrolyte. Previous studies often used carbonate ester electrolyte in RT Na–S batteries, which went through a slow ‘solid–solid’ conversion in the sulfur cathode and achieved stable cyclability. The sulfur content was usually around only 50%, however, which was too low for practical applications. To increase the sulfur content, we study two major sulfur species and their electrochemical behavior in two dominant nonaqueous electrolyte systems, carbonate ester electrolyte and ether electrolyte. The sulfur-rich sample (155S) has high sulfur content (72%) with most of sulfur on the surface of the cathode host, which suffers from severe nucleophilic addition or substitution reactions between the nucleophilic polysulfide anions and carbonate ester solvents, thus causing a rapid capacity fading. On the other hand, sulfur in the pores (300S) features with low sulfur content (44%) manifesting the ‘solid–solid’ reaction in both carbonate ester and ether electrolytes. To improve cyclability of the 155S, tetraethylene glycol dimethyl ether (TEGDME) electrolyte is applied, in which dissolved polysulfides can stably exist. However, the dissolved polysulfides cause serious shuttle effect, which also results in poor cyclability. To address this problem, NaNO_3_ additive is applied to form a stable SEI to confine the deposition of nonconductive Na_2_S on sodium anode. Overall, the resulting 155S electrode not only achieves high sulfur content (72%), but also stable cyclability with 483 mAh g^−1^ reversible capacity and 362 Wh kg^−1^ energy density. The ‘solid–liquid’ conversion in ether electrolyte provides pathways for ionic conduction in the sulfur-rich cathode, shedding light on how to achieve practical application of the RT Na–S batteries.

## Experimental

### Chemicals

Analytical-grade zinc nitrate hexahydrate (Zn(NO_3_)_2_·6H_2_O) and 2-methylimidazole were obtained from Sigma-Aldrich.

### Preparation of Pristine Carbon Host

In a typical procedure, Zn(NO_3_)_2_·6H_2_O (6.4 mmol) and 2-methylimidazole (3.2 mmol) were dissolved in 80 mL methanol and stirred for 5 min. After aging for 12 h, the as-obtained precipitates were centrifuged, washed with ethanol several times, and dried in vacuum at 70 ℃ overnight. The as-obtained purple powder was annealed at 1000 °C for 5 h with a heating rate of 2 °C min^−1^ in N_2_.

### Preparation of the 155S

The obtained carbon host was mixed with sulfur powder in the mass ratio of 1:3. The mixture was sealed in a glass tube and annealed at 155 °C for 12 h. The resultant powder was denoted as 155S.

### Preparation of the 300S

Pristine carbon host was mixed with sulfur powder in the mass ratio of 1:3. The mixture was sealed in glass tube and annealed at 155 °C for 12 h, at which solid S turned into liquid S and evenly mixed with carbon host. The temperature was then further increased to 300 °C with a holding time of 5 h. At 300 °C, the liquid S was boiling and easily got into the inner pore of carbon host.

### Characterization and Measurements

The morphologies of the samples were investigated by SEM (JEOL 7500) and STEM (JEOL ARM-200F, 200 keV). Powder X-ray diffraction (XRD) patterns were collected by powder XRD (GBC MMA diffractometer) with Cu Kα radiation at a scan rate of 2.5° min^−1^. X-ray photoelectron spectroscopy (XPS) measurements were carried out using Al Kα radiation and fixed analyzer transmission mode: the pass energy was 60 eV for the survey spectra and 20 eV for the specific elements.

### Preparation of Battery Cells

The electrochemical tests were conducted by assembling 2032 coin-type half-cells in an argon-filled glove box. For the Li–S batteries, the slurry was prepared by fully mixing 80 wt% cathode materials, 10 wt% carbon black, and 10 wt% polyvinylidene difluoride (PVDF) in an appropriate amount of *N*-methyl-2-pyrrolidone (NMP) via a planetary mixer (KK-250S). Then, the obtained slurry was pasted on Al foil, which was followed by drying at 60 °C in a vacuum oven overnight. The working electrode was prepared by punching the electrode film into disks 1.0 cm diameter. The loading of sulfur was around 2 mg_s_ cm^−1^ in each electrode. Lithium foil was employed as both reference and counter electrodes. The electrodes were separated by a polypropylene separator, which was 2.8 cm in diameter. The electrolyte consisted of 1 M lithium bis(trifluoromethanesulfonyl)imide (LiTFSI) and 2 wt% LiNO_3_ in 1,3-dioxolane: 1,2-dimethoxyethane = 1:1 v/v. About 40 μL of electrolyte was added to each coin cell via a microliter syringe.

For the RT Na–S batteries, the slurry was prepared by fully mixing 80 wt% cathode materials, 10 wt% carbon black, and 10 wt% carboxymethyl cellulose (CMC) in an appropriate amount of water via a planetary mixer (KK-250S). Then, the obtained slurry was pasted on Cu foil, which was followed by drying at 60 °C in a vacuum oven overnight. The working electrode was prepared by punching the electrode film into disks 1.0 cm diameter. The loading of sulfur was around 2 mg s cm^−1^ in each electrode. Sodium foil was employed as both reference and counter electrodes. The electrodes were separated by a glass fiber separator. The carbonate ester electrolyte was 1 M NaClO_4_ dissolved in ethylene carbonate (EC): diethyl carbonate (DEC) = 1:1 v/v with the addition of 5% fluoroethylene carbonate (FEC). The ether electrolyte was 1 M NaClO_4_ dissolved in tetraethylene glycol dimethyl ether (TEGDME) with/without the addition of 0.2 M NaNO_3_ additive. About 40 μL of electrolyte was added to each coin cell via a microliter syringe.

### Electrochemical Testing of Battery Cells

The electrochemical performance of the batteries was tested on a LAND Battery Tester with a voltage window of 1.7–2.8 V for the Li–S batteries and 0.8–2.8 V for the RT Na–S batteries. All the capacities of cells were normalized based on the weight of sulfur. Cyclic voltammetry (CV) was performed using a Biologic VMP-3 electrochemical workstation. Calculation of the lithium-ion diffusion coefficient: In order to explore the lithium diffusion properties, we performed cyclic voltammetry (CV) measurements under different scanning rates. All the cathodic and anodic peak currents were linear with respect to the square root of the scan rate, from which the lithium diffusion performance could be estimated using the classical Randles–Sevcik equation:

1 $$I_{p} = \left( {2.69 \times 10^{5} } \right)n^{1.5} AD^{0.5} C\nu^{0.5}$$

where *I*_p_ is the peak current, n is the charge transfer number, *A* is the electrode area, *D* is the lithium-ion diffusion coefficient, *C* is the Li^+^ concentration, and *ν* is the scan rate.

## Results and Discussion

### Characterization of the 155S and 300S

**Fig. 1:**
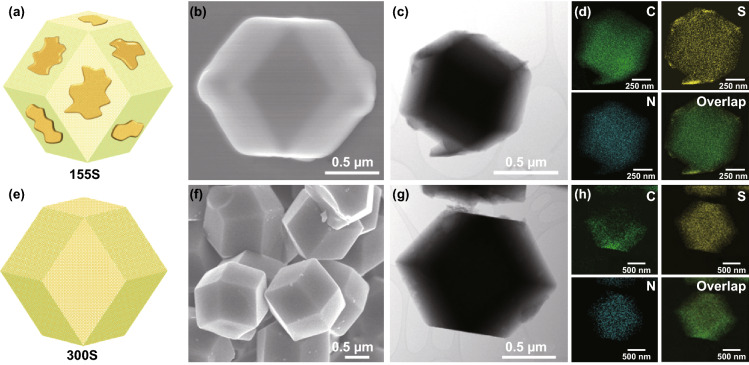
**a** Schematic illustration of the 155S. **b** SEM image of the 155S. **c** STEM image of the 155S. **d** EDS mappings of the 155S. **e** Schematic illustration of the 300S. **f** SEM image of the 300S. **g** STEM image of the 300S. **h** EDS mappings of the 300S

Figure 1a presents a schematic illustration of the 155S sample, where the sulfur mainly covers the surface of carbon host. When conducting the scanning electron microscopy (SEM), the 155S sample had to be prepared with Pt spray to improve the conductivity and was evenly dispersed on a silicon wafer. Even so, the resulting image does not look very clear because the nonconductive sulfur mainly covered the surface of the carbon host, affecting resolution (Fig. 1b). In comparison, the 300S powder was simply pasted on conductive plastic for its SEM image (Fig. 1f). The resulting image of 300S is much clearer than that of 155S, indicating that the 300S has better conductivity than the 155S. According to the energy-dispersive X-ray spectroscopy (EDS) mapping, the sulfur on the 155S has strong intensity on the surface, while the sulfur in the 300S is evenly dispersed throughout the whole particle. These findings demonstrate that the surface of the 155S sample is mainly composed of sulfur, while the majority of the sulfur in the 300S is stored in the pores of the cathode host.

**Fig. 2:**
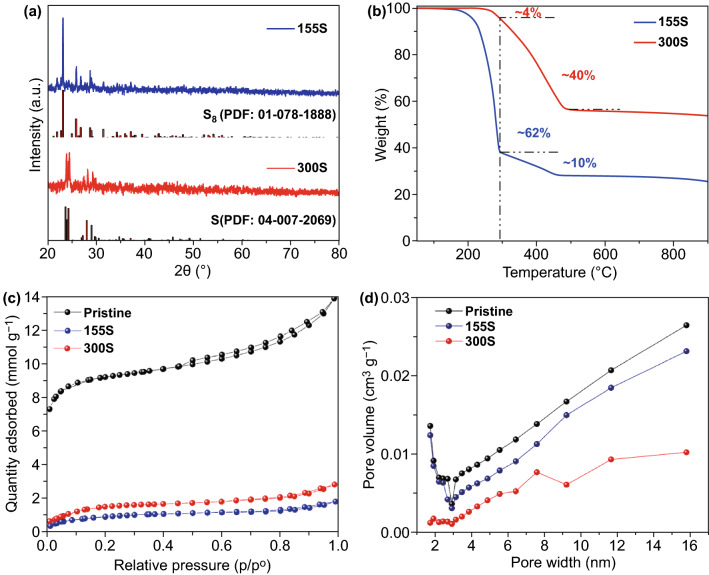
**a** XRD patterns of the 155S and 300S. **b** TGA curves of the 155S and 300S. **c** BET isotherms of the pristine carbon host, 155S, and 300S. **d** Pore dispersions of the pristine carbon host, 155S, and 300S

To study the phases of 155S and 300S, powder XRD is employed. There are two different patterns for the 155S and the 300S, indicating they contain different types of sulfur. For the 155S, strong peaks at 23.1°, 25.8°, 26.7°, and 28.7° are indexed to the (111), (013), (311), and (313) planes of crystalline S_8_ (PDF: 01-078-1888), respectively (Fig. 2a) [25]. This result is in accordance with most studies on Li–S batteries, in which cathode hosts are sulfurated at 155 °C [40–42]. In the case of the 300S, strong peaks at 23.8°, 24.1°, 24.4°, and 28.1° are indexed to the (212), (130), (012), and (132) planes of crystalline S (PDF: 04-007-2069), respectively. Some studies also sulfurated their cathode hosts at 300 °C with residual S_8_ [20]. To remove the residual S_8_, it is found to be advisable to extend the holding time at 300 °C to 5 h. The thermogravimetric analysis (TGA) curve shown in Fig. 2b confirms the different amounts of S contained in the 155S and 300S, with 72 and 44 wt%, respectively. Significantly, the S loading ratio in the 155S is much higher compared with the 300S. There are two main states of fast weight loss with rising temperature in the two samples. Sulfur on the surface is easily evaporated in the low-temperature state, while the sulfur stored in the porous structure of the carbon host requires more energy to evaporate out. In the case of the 155S, crystalline S_8_ on the surface of the carbon host sublimes at a relatively low temperature of ~ 290 °C, which accounts for ~ 62 wt% of the total mass. Then, a small amount of sulfur confined in the pore structure evaporates when the temperature increases from 290 to 460 °C with a weight loss of ~ 10 wt%. In comparison, the 300S goes through a slight weight loss (~ 4 wt%) at low temperature (below 290 °C). As the temperature further increases to 460 °C, a large amount of sulfur (~ 40 wt%) evaporates out, suggesting that the 300S is mainly composed of sulfur in the pore. Regarding the Brunauer–Emmett–Teller (BET) analysis, a large specific surface area is confirmed in pristine carbon host with 641.3 m^2^ g^−1^. After sulfuration at 155 °C, the resulting 155S shows a much lower specific surface area with only 54.2 m^2^ g^−1^, because the implanted sulfur significantly increases the mass of sample. A similar profile is also found for the 300S sample, where the specific surface area slightly increases to 89.3 m^2^ g^−1^ after most of the surface sulfur evaporated out. The hierarchical pore structure can be evaluated by the pore size distributions in Fig. 2d. Mesopores are pores of internal width between 2 and 50 nm, while micropores are defined as pores with internal diameters of less than 2 nm. Based on the BET test, we calculated the pore size distribution of the pristine carbon host. The pore volume of mesopores is 0.1511 cm^3^ g^−1^ while the pore volume of micropores is 0.0367 cm^3^ g^−1^ (Fig. S1). The total pore volume of mesopores and micropores in the pristine carbon host is 0.1878 cm^3^ g^−1^. After sulfur impregnation, the 155S and 300S samples are observed to have pore volume of 0.1545 cm^3^ g^−1^ and 0.0658 cm^3^ g^−1^, respectively. The relatively low pore volume in the 300S suggests that sulfur was implanted into the pore structure of the carbon host, which dramatically decreased the pore volume. Based on above studies, we find that the 155S had a higher sulfur proportion (72%), which is mainly contributed by the S_8_ dispersed on the surface, whereas the 300S had only 44% sulfur content with most of the S stored in the pores of the cathode host.

To investigate the cycling performance of the 155S and 300S in Li–S batteries, we applied a common ether electrolyte (1.0 M lithium bis(trifluoromethanesulfonyl) imide (LiTFSI) and 0.2 M LiNO_3_ in 1,3-dioxolane: 1,2-dimethoxyethane = 1:1 v/v) in Li–S battery cells [43, 44]. Lithium foil functioned as the anode material, and Celgard 2500 was chosen as the separator. The electrode with the 155S cathode delivers a reversible capacity of 491 mAh g^−1^ after 200 cycles based on the mass of sulfur (72%). In comparison, the cathode with the 300S (44% S) realizes a high capacity of 696 mAh g^−1^ (Fig. S2). Good conductivity and sulfur utilization can usually be achieved with relatively low sulfur content, leading to high capacity in Li–S batteries [39, 45]. The discharge/charge curves of the 155S electrode show a dual discharging platform at around 2.3 and 2.1 V, corresponding to a ‘solid–liquid’ conversion from solid S_8_ to liquid LiPSs and from liquid LiPSs to solid Li_2_S, respectively [46, 47]. However, the 300S electrode shows only one discharging platform at around 2.1 V, which is a ‘solid–solid’ conversion from solid S to solid Li_2_S [48]. The ‘solid–solid’ conversion usually takes place in molecular sulfur (S_2-4_), atomic sulfur (SPAN as a representative), or in electrolytes with non- or sparingly solvated PSs with a conventional C/S composite cathodes [28–32]. Our finding makes it manifest that the ‘solid–solid’ conversion can also take place in crystalline S coupled with electrolyte, when S is confined in the pores of the cathode host. To further visualize this phenomenon, we disassembled the battery cells after cycling. The electrolyte in the 155S electrode has a yellow color, indicating the presence of dissolved LiPSs. In comparison, the electrolyte in the 300S electrode remains colorless, suggesting the absence of dissolved LiPSs (Fig. S3). To study the lithium-ion diffusion in the 155S and 300S, CV profiles at were collected at different scanning rates (Fig. S4). The lithium diffusion coefficients in the two types of sulfur were calculated based on the Randles–Sevcik equation at a series of CV scanning rates (Fig. S5) [49]. Slopes in the 300S are much steeper than those in the 155S, indicating a better diffusion of lithium ions in the 300S. Although dissolved LiPSs in the 155S can serve as intrinsic redox mediators to activate deactivated sulfur and increase the utilization of sulfur, the high sulfur content and poor contact between carbon host and surface sulfur in the 155S inevitably cause poor conductivity and sluggish kinetics.

### Cycle Performance in Carbonate Ester Electrolyte

**Fig. 3:**
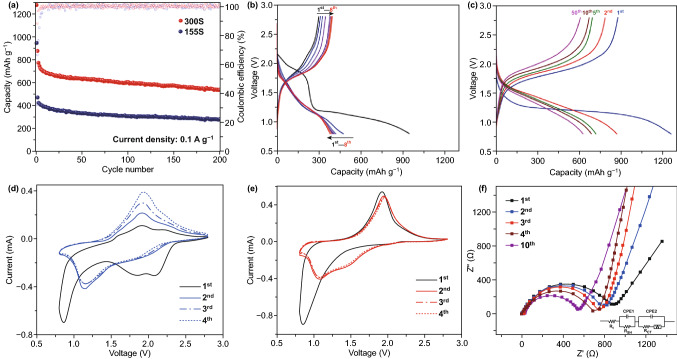
**a** Electrochemical cycling performances of the 155S and 300S samples at 0.1 A g^−1^ in carbonate-based electrolyte. **b** Discharge/charge curves of the 155S at 0.1 A g^−1^. **c** Discharge/charge curves of the 300S at 0.1 A g^−1^. **d** CV curves for the 155S at 0.1 mV s^−1^. **e** CV curves for the 300S at 0.1 mV s^−1^. **f** EIS spectra of the 155S electrode, with the inset equivalent circuit used to interpret the results

With respect to the RT Na–S batteries, we applied a common carbonate ester electrolyte (1 M NaClO_4_ in EC/DEC = 1:1 v/v with 5% FEC additive), sodium foil as the anode material, and glass fiber as separator. All the battery cells were tested in the voltage range from 0.8 to 2.8 V. As shown in Fig. 3a, the 155S electrode delivers a reversible capacity of 279 mAh g^−1^ after 200 cycles based on the mass of sulfur (72%). In comparison, the cathode with the 300S (44% S) realizes a high capacity of 535 mAh g^−1^. Notably, the initial CE of the 155S (31.7%) is much lower than that of the 300S (68.6%). The 300S undergoes a complex activation process in carbonate ester electrolytes during the initial discharge, involving electrolyte decomposition and nucleophilic reactions between carbonate ester solvents and polysulfides [25]. These irreversible reactions lead to the formation of solid–electrolyte interphase (SEI) on the Na anode and cathode electrolyte interphase (CEI) on the C/S cathode and result in large initial discharge capacity [19]. However, these reactions are irreversible in the charge causing poor initial Coulombic efficiency. For the 155S, in the first discharge, S_8_ on the surface of carbon host turns into long-chain polysulfides at around 2.0 V but the resulting polysulfides have side reaction with carbonate ester solvents [27]. As a result, the 2.0 V platform is irreversible in following discharge. In the following cycles, small amount of the sulfur stored in the pores of cathode host undergoes a ‘solid–solid’ reaction with sodium, resulting in a very low initial CE and poor reversible capacity. Regarding rate performance, the 155S electrode exhibits performances of 290, 187, and 142 mAh g^−1^ at 0.1, 0.5, and 1.0 A g^−1^, respectively. In comparison, the 300S electrode displays relatively high capacities of 540, 345 and 243 mAh g^−1^ at 0.1, 0.5 and 1.0 A g^−1^, respectively, showing the benefit of confined S in carbonate ester electrolyte (Fig. S6). The charging capacity is gradually increased from the first to the 8th cycle, indicative of sluggish kinetics in the 155S selectrode (Fig. 3b). In comparison, the 300S electrode does not show a platform around 2.0 V in the initial discharge, indicating sulfur confined in the pores skips the conversion toward long-chain polysulfides. Thus, the 300S electrode has a much higher initial CE than the 155S [25]. In the following cycles, the 300S electrode shows similar charge–discharge profiles to the 155S because it is going through the same ‘solid–solid’ conversion from polysulfides to Na_2_S (Fig. 3c). The cyclic voltammetry (CV) profiles also show similar behaviors in Fig. 3d, e. There is a prominent peak centered at 2.2 V during the first cathodic scan for the 155S, which corresponds to the side reaction between surface polysulfides and carbonate ester solvent. In the following cathodic scans, a repeatable reduction peak appears at 1.1 V for the 155S and 300S, which corresponds to the formation of Na_2_S. The highly repeatable scans without current attenuation indicate a reversible reaction mechanism with high capacity retention in this system. According to the electrochemical impedance spectra (EIS) in Fig. 3f, the impedance of the 155S electrode decreases with cycling. After ten cycles, the impedance (544 Ω) is much lower than in the initial state (904 Ω), indicating that the conductivity of 155S will be gradually improved by cycling. Overall, the 300S electrode (44% S) shows high capacity in carbonate ester electrolyte because the loaded sulfur is confined in the pores, which cannot directly contact with solvents and avoid the occurrence of the side reactions [25]. In comparison, although the 155S has a high sulfur content (72%), most of sulfur will need to be dispersed on the surface of cathode host. It shows poor cyclability because of severe side reactions between nucleophilic polysulfide anions and carbonate ester solvents. Therefore, it is necessary to develop another type of nonaqueous electrolyte, ether electrolyte, for the S-rich cathode in the RT Na–S batteries.

### Cycle Performance in Ether Electrolyte

**Fig. 4:**
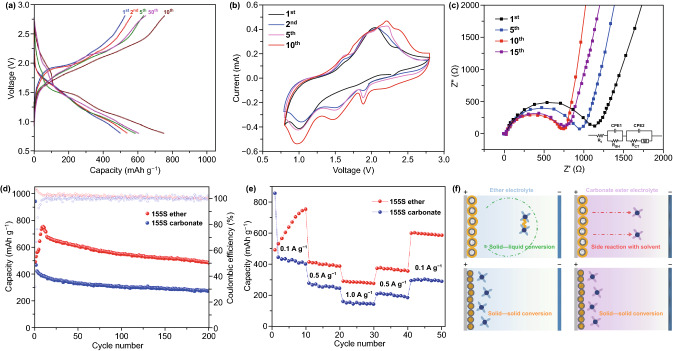
**a** Discharge/charge curves of the 155S in ether electrolyte with NaNO_3_ additive. **b** Corresponding CV curves of the 155S in ether electrolyte with NaNO_3_ additive. **c** Corresponding EIS spectra of the 155S in ether electrolyte with NaNO_3_ additive. **d** Cycling performances of the 155S at 0.1 A g^−1^ based on the mass of sulfur in ether and carbonate electrolyte. **e** Rate performance of the 155S at 0.1 A g^−1^ based on the mass of sulfur in ether and carbonate electrolyte. **f** Schematic illustrations of the mechanisms in ether and carbonate electrolyte for the sulfur on the surface (155S) and the sulfur in the pore of cathode host (300S)

As learned from the Li–S batteries, NaNO_3_ additive is promising for limiting the shuttle effect like LiNO_3_. We tried three types of solvent, but only TEGDME could dissolve NaNO_3_ (Fig. S7), so we used TEGDME with 1 M NaClO_4_ sodium salt and 0.2 M NaNO_3_ additive as our chosen electrolyte. As shown in Fig. 4a, the 155S electrode was tested in TEGDME under the same conditions as our previous test in carbonate ester electrolyte. The high sulfur content and thick cathode material in each electrode cause poor conductivity and sluggish kinetics, so that it takes several cycles to activate all of the sulfur. After the sulfur was fully reacted with sodium a platform above 1.9 V appears in the tenth cycle during discharge. According to previous studies, this platform corresponds to the production of liquid Na_2_S_*x*_ (4 < *x* ≤ 8) [50]. As the voltage drops to around 1.6 V, another platform corresponding to the conversion from liquid Na_2_S_*x*_ to solid Na_2_S appeared. According to the CV profile, there are three cathodic peaks at 1.9, 1.5, and 1.0 V, respectively. The peak around 1.9 V corresponds to the formation of liquid Na_2_S_*x*_, while the following peak at 1.5 V corresponds to Na_2_S_4_, which further splits into Na_2_S at 1.0 V [51]. Compared to carbonate ester electrolyte, the 155S electrode tested in ether electrolyte shows very specific peaks for each conversion. Regarding the conductivity, the impedance of 155S electrode is reduced from 1140 to 743 Ω in the first ten cycles, indicating that sulfur is gradually activated in each cycle. After 15 cycles, the impedance slightly increases to 770 Ω as nonconductive Na_2_S was deposited on the sodium foil.

With respect to the cycling performance (Fig. 4d), the 155S electrode exhibits improved cyclability with capacity of 483 mAh g^−1^ in ether electrolyte at 0.1 A g^−1^, compared to its counterpart of 279 mAh g^−1^ in carbonate ester electrolyte, showing the promise of ether electrolyte for application in the RT Na–S batteries. As the current density further increased to 3.0 A g^−1^, the 155S also delivers a stable capacity of 123 mAh g^−1^ after 300 cycles (Fig. S8). The additive of NaNO_3_ in TEGDME electrolyte shows an improved cyclability for the 155S electrode. To further study the impact of the dose of NaNO_3_ additive on cycle performance, we tested the 155S electrodes in TEGDME electrolytes with different doses of NaNO_3_ additive. Under same conditions, the 155S electrodes deliver reversible capacity of 395, 476, and 561 mAh g^−1^ in 0.05, 0.1, and 0.2 M NaNO_3_ additive, respectively, suggesting that reversible capacity improves along with the increase in NaNO_3_ (Fig. S9). The 155S is composed of large amount of sulfur (72% S) and dispersed on the surface of carbon host, which inevitably leads to poor conductivity and sluggish kinetics. With the increase in S content in electrode, the sluggish kinetics will be more serious. The 155S electrodes with S content of 1, 2, and 3 mg cm^−2^, respectively, exhibit 667, 562, and 490 mAh g^−1^ after 40 cycles at the current density of 0.1 A g^−1^ (Fig. S10). During initial several cycles, solvated cation Na^+^ slowly reacts with sulfur from the surface to the core, resulting in the formation of dissolved polysulfides. As sulfur gradually activated, the accessible capacity is also increased. In comparison, the 300S is mainly stored in the pore of carbon host with S content of 44%. The carbon framework offers high conductivity to S ensuring a stable reaction kinetics for the 300S. When S content increasing from 1 and 2 mg cm^−2^ to 3 mg cm^−2^, the 300S electrodes exhibit reversible capacity of 446, 377, and 301 mAh g^−1^ after 40 cycles at the current density of 0.1 A g^−1^, respectively. For the 155S, long-chain polysulfides spontaneously shuttle to Na anode during discharge. Part of the polysulfides react with Na anode resulting in Na_2_S deposit. This process consumes active materials but does not contribute to discharging capacity. During charge, part of Na_2_S deposit is oxidized into polysulfides while polysulfides will shuttle back to cathode under the influence of external forces, which consumes additional energy and causes high charging capacity resulting in the Coulombic efficiency over 100%. To meet the practical expectations, we tried to decrease the electrolyte amount from 20 to 4.5 μL mg s^−1^ (Fig. S11). The 155S electrode delivers reversible capacity of 320 mAh g^−1^ with 4.5 μL mg s^−1^ ether electrolyte compared to the counterpart of 641 mAh g^−1^ with 20 μL mg s^−1^. By decreasing the E/S ratio, the mass and cost of battery cell can effectively reduce. However, the decrease in electrolyte can also bring up with other issues including poor permeability, low S utilization and sluggish kinetics. The concentration of dissolved polysulfides increases along with the decrease in electrolyte amount. As a result, large amount of S species cannot be electrochemically converted fast enough to keep up with the charging/discharging rate to deliver reversible capacity, and ‘dead’ S will be accumulated on the electrode surface over cycling [52]. The resulting ‘solid–liquid’ conversion path represents an alternative to the ‘solid–solid’ conversion. Moreover, the 155S electrode also presents promising rate performance, delivering reversible capacity of 586, 361, and 275 mAh g^−1^ at current densities of 0.1, 0.5, and 1.0 A g^−1^, respectively (Fig. 4e). In contrast, the 300S electrode in ether electrolyte shows similar voltage–capacity profile as the one in carbonate ester electrolyte (Fig. S12), emphasizing the same ‘solid–solid’ conversion in ether electrolyte. However, according to previous study, carbonate ester electrolyte usually delivers higher and more stable capacity than the ether counterpart, which is related to the formation of SEI layer [53, 54]. Besides, the FEC additive can form a protective SEI layer achieving better cycling performance (Fig. S13). These factors make the 300S better performance in carbonate ester electrolyte than in ether electrolyte. Figure 4f summarizes the mechanisms of sulfur on the surface (155S) and in the pores of cathode host (300S) when working in ether and carbonate ester electrolytes, respectively. In the ether electrolyte, the 155S directly reacts with solvated cation Na^+^ resulting in dissolved polysulfides (solid–liquid conversion). The 300S is encapsulated in carbon host and converted into Na_2_S by ion exchange (solid–solid conversion). In carbonate ester electrolyte, the surface S in the 155S suffers from severe nucleophilic addition or substitution reactions between the nucleophilic polysulfide anions and carbonate ester solvent, thus causing a serious side reaction and rapid capacity fading [55]. In contrast, the encapsulated 300S does not directly contact with carbonate ester solvent, which can deliver superior reversible capacity via ‘solid–solid’ conversion. The atomic diameter of Na is larger than that of Li, which means the molecular size of sodium polysulfides is larger than the counterparts of lithium polysulfides. The bigger size of sodium polysulfides makes them more difficult to escape from carbon host than the lithium polysulfides. Besides, S_8_ was proven to exceptionally undergo a ‘solid–solid’ conversion for Li–S batteries in the absence of micro/mesoporous structure [56]. Due to the function of high-concentrated lithium salt, a dense CEI was constructed on the surface of S active material after the first cycle. The CEI layer transited the subsequent S electrochemistry from a typical ‘solid–liquid’ conversion to ‘solid–solid’ conversion. Therefore, we believe the CEI layer may have similar function in RT Na–S batteries by separating the sodium polysulfide from outside electrolyte. Combined with our experimental results, S_8_ is applicable to conduct ‘solid–solid’ conversion in mesoporous host.

In the ‘solid–liquid’ conversion, dissolved polysulfides will spontaneously shuttle to the sodium anode, however, and turn into nonconductive Na_2_S, causing serious capacity fading. As shown in Fig. S14, the voltage–capacity profile shows that discharging capacity generated from the voltage above 2.0 V is much lower than the one with NaNO_3_, indicating that NaNO_3_ offers effective confinement of dissolved the polysulfides to prevent the shuttle effect [53]. Without NaNO_3_ additive, the 155S electrode only achieves relatively low capacity of 395, 264, and 212 mAh g^−1^, respectively. When the weight of binder, carbon black, and cathode host is counted in total mass, the resulting energy density of the 155S (72% S) electrode reaches 362 and 260 Wh kg^−1^ in TEGDME with/without NaNO_3_ additive (Fig. S15).

### Characterization of Na Anode

**Fig. 5:**
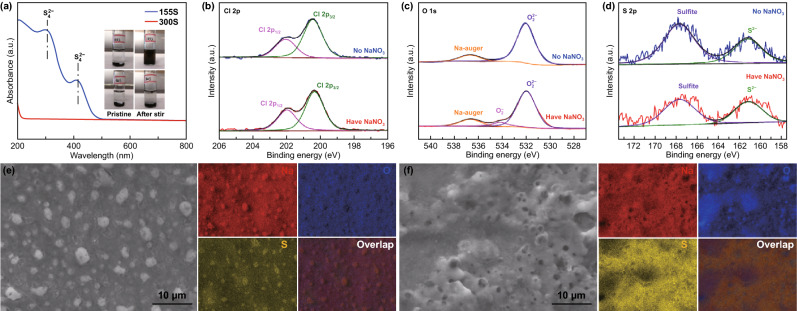
**a** Photographs (inset) and UV–Vis spectra of 155S and 300S dispersed in ether electrolyte and stirred with Na metal for 5 min. **b** XPS spectra of the Na metal after cycling with the 155S electrode in ether electrolyte for 200 cycles for the Cl 2*p*. **c** XPS spectra of the Na metal after cycling with the 155S electrode in ether electrolyte for 200 cycles for the O 1 s. **d** XPS spectra of the Na metal after cycling with the 155S electrode in ether electrolyte for 200 cycles for the S 2*s* regions. **e** SEM image and EDS mappings of the Na metal after 200 cycles with the 155S in ether electrolyte with NaNO_3_ additive. **f** SEM image and EDS mappings of the Na metal after 200 cycles with the 155S in ether electrolyte without NaNO_3_ additive

As shown in Fig. 5a, we added the 155S and 300S powders into ether electrolyte at the same ratio of cathode/electrolyte as in the tested battery cell. A small amount of bulk sodium metal was dropped into the mixture. These two bottles of with 155S and 300S had the same color in the pristine state. After stirring for 5 min in an Ar-filled glove box, the 155S mixture turned brown, while the 300S remained transparent. According to the ultraviolet–visible (UV–Vis) spectroscopy, S_4_^2−^ was detectable from the 155S electrolyte after stirring, whereas there were no obvious peaks from the transparent 300S electrolyte [57]. This result visualizes the ‘solid–liquid’ and ‘solid–solid’ conversions, in which the sulfur on the surface of the cathode host will dissolve into the electrolyte after reacts with sodium, while the sulfur stored in the pores of the cathode host will be trapped in the porous structure, even after reacting with sodium. Since dissolved polysulfides can spontaneously shuttle to the sodium anode, the side reactions will dramatically reduce the reversible capacity. The reversible capacity is significantly improved, however, with the addition of NaNO_3_, indicating that the shuttle effect is confined. To find out the reason, we performed X-ray photoelectron spectroscopy (XPS) on the sodium foil after 200 cycles in ether electrolyte with/without NaNO_3_ additive. In the Cl 2p region (Fig. 5b), Cl 2*p*_1/2_ and Cl 2*p*_3/2_ are located at 202.2 and 200.6 eV, respectively [58]. The Cl element originated from the NaClO_4_ sodium salt, and therefore, it is detectable in both samples. With respect to the O region, there is a Na-Auger peak at 536.9 eV for two samples [59]. A peak for O_2_^2−^ at 532.2 eV is also detected in the two samples [60]. Notably, a new peak situated at 534.1 eV was found in the sample with NaNO_3_, which corresponds to O_2_^−^ [60]. These new bonds may have potential impact towards confining polysulfide shuttling. In the S 2p region, the peak located at 161.2 eV is corresponding to S^2−^ [61]. Another peak situated at 167.8 eV is in accord with sulfite [62]. Obviously, these two peaks in the sample without NaNO_3_ additive attain a stronger intensity than in the one with NaNO_3_ additive, indicating that O_2_^−^ is promising to confine the polysulfides and prevent the shuttle effect. SEM images and EDS mappings were further employed to visualize the confinement of polysulfides. As shown in Fig. 5e, the surface of the sodium foil is dotted with evenly dispersed bright particles after cycling in the electrolyte with NaNO_3_. According to the EDS mapping, the isolated particles are composed of Na and S, which should be assigned as Na_2_S according to the XPS result [63]. In the cycling process, SEI layer is formed on the sodium foil after the first discharge. The SEI will be reconstructed during sodium insertion/extraction [64–66]. Our study shows that the Na–O-rich SEI layer is promising to limit the dissolved polysulfides reacting with sodium. And the NaNO_3_ additive is helpful to form the Na–O-rich SEI layer on the sodium foil, confining the growth of Na_2_S. As a result, the Na_2_S is isolated in small particles. In comparison, after cycling the sodium foil without NaNO_3_ is detected with a thick layer of Na and S. Although a Na–O-rich SEI layer is also formed, it is not evenly dispersed on the sodium foil and hardly preventing the side reactions between Na and S. Figure 5f shows a mass of Na_2_S deposit on Na anode. During discharge, long-chain polysulfides freely shuttle and spontaneously react with Na. This process cannot contribute to discharging capacity but consumes active materials. During charge, part of Na_2_S is oxidized into long-chain polysulfides and the long-chain polysulfides will shuttle back to the cathode under the influence of external forces. This process causes much more charging capacity than the counterpart with NaNO_3_ additive. According to previous study, LiNO_3_ participated in the formation of a stable passivation film which can effectively suppress the redox shuttle of the dissolved lithium polysulfides on Li anode [55]. The NaNO_3_ additive shares a similar function as LiNO_3_ in RT Na–S batteries that forms a Na–O-rich SEI layer to limit the redox shuttle of the dissolved sodium polysulfides.

## Conclusions

Overall, we have successfully increased sulfur utilization from ~ 50 to 72% via a ‘solid–liquid’ conversion in RT Na–S batteries. The mechanisms of two types of sulfur, sulfur in the pores (300S) and sulfur on the surface (155S) and of the host, have been studied in two typical nonaqueous electrolytes, respectively. The 300S encapsulated in carbon host does not directly contact with solvents; thus it performs reversible ‘solid–solid’ conversion in both ether and carbonate ester electrolytes. In comparison, the 155S goes through ‘solid–liquid’ conversion from S_8_ to dissolved polysulfides in ether electrolyte but suffers from severe side reactions between the nucleophilic polysulfide anions and the solvent in carbonate ester electrolyte. Moreover, we have also investigated the function of NaNO_3_ additive that forms a Na–O-rich SEI layer confining the deposition of Na_2_S on Na anode in ether electrolyte. As a result, the 155S electrode has not only high sulfur content (72%), but also stable cyclability with reversible capacity of 483 mAh g^−1^ and energy density of 362 Wh kg^−1^ after 200 cycles. The ‘solid–liquid’ conversion in ether electrolyte is an effective pathway for sulfur-rich cathode, shedding light on achieving high-performance cathode for practical applications of RT Na–S batteries.

## Supplementary Information

Below is the link to the electronic supplementary material.

Supplementary file1 (PDF 968 kb)
