# Supplementary material for: Understanding Sulfur Redox Mechanisms in Different Electrolytes for Room-Temperature Na–S Batteries
Source: Nanomicro Lett. 2021 May 4;13:121. doi: 10.1007/s40820-021-00648-w (PMC8096878; doi:10.1007/s40820-021-00648-w)
Supplement: Supplementary file 1 — Supplementary file1 (PDF 968 kb) [file 40820_2021_648_MOESM1_ESM.pdf]

Supporting Information for

Understanding Sulfur Redox Mechanisms in Different Electrolytes for Room-Temperature Na-S Batteries

Hanwen Liu[1], Weihong Lai[1], Qiuran Yang[1], Yaojie Lei[1], Can Wu[1], Nana Wang[1], Yunxiao Wang[1,] *, Shulei Chou[1], Hua Kun Liu[1], Shi Xue Dou[1]

[1]Institute for Superconducting and Electronic Materials, Australian Institute of Innovative Materials, University of Wollongong, Innovation Campus, Squires Way, North Wollongong, NSW 2500, Australia

*Corresponding author. E-mail: yunxiao@uow.edu.au (Yunxiao Wang)

Supplementary Figures

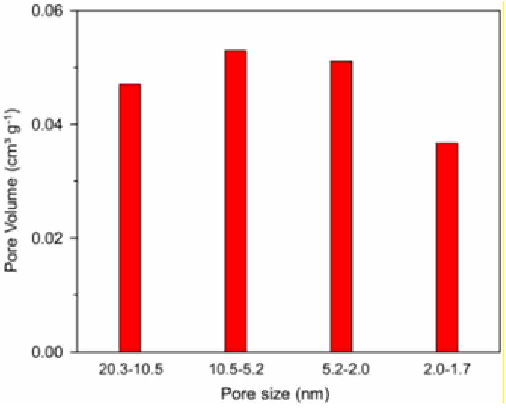

Fig. S1 Pore distribution of pristine carbon host

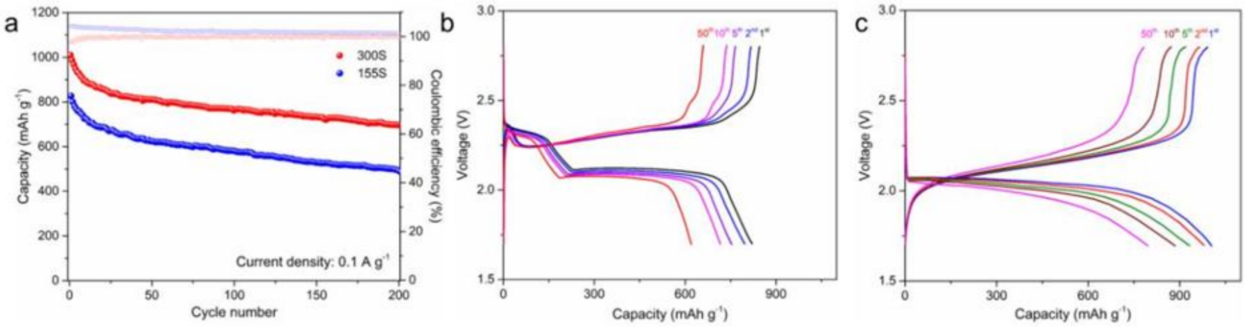

Fig. S2 (**a**) Cycling performances of the Li-S batteries based on 155S and 300S at 0.1 A g^{-1}; Discharge/charge curves of (**b**) the 155S and (**c**) the 300S at 0.1 A g^{-1}

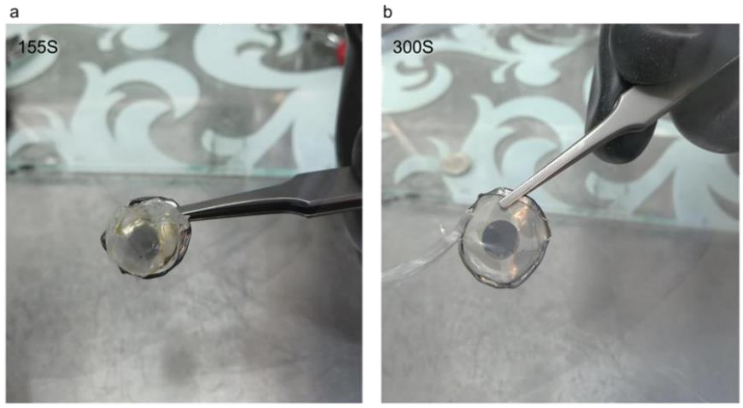

Fig. S3 Images of disassembled battery cells with (**a**) 155S electrode and (**b**) 300S electrode

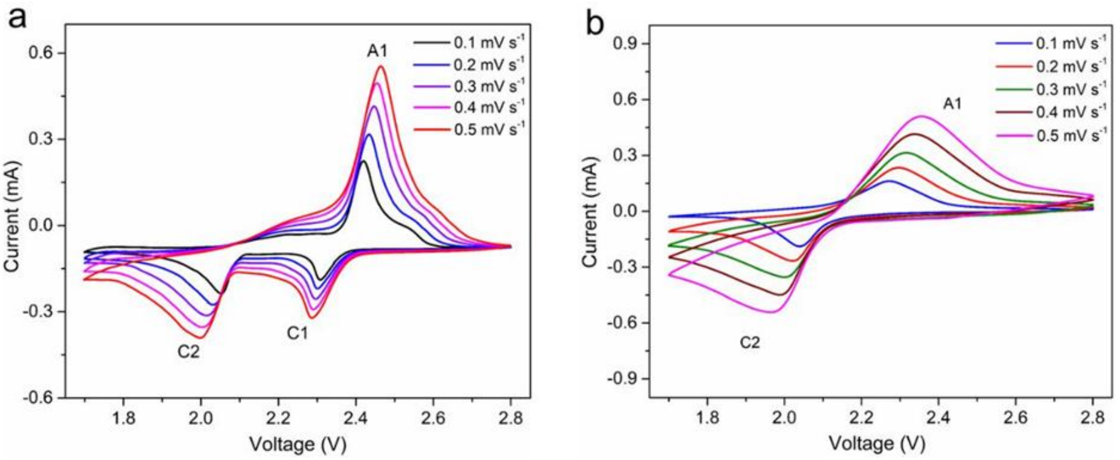

Fig. S4 CV curves for (**a**) the 155S and (**b**) the 300S at different scan rates

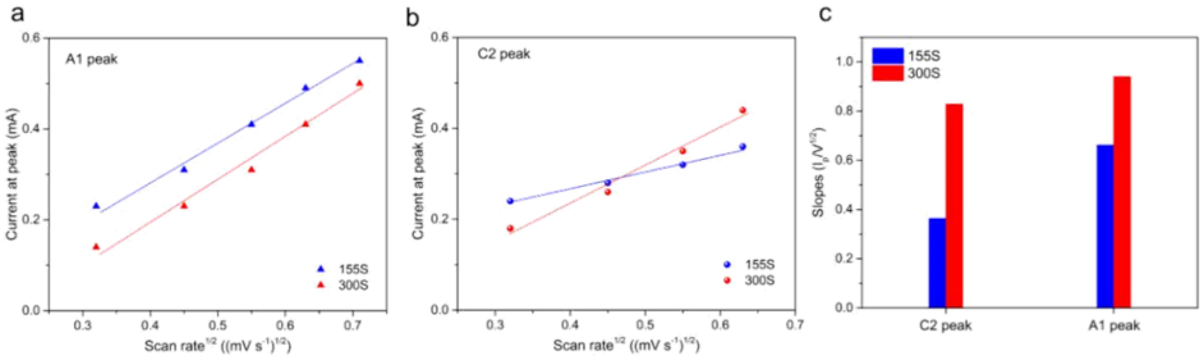

Fig. S5 Linear fits of the $I\text{p}/v^{1/2}$ for (**a**) the A1 and (**b**) the C2 peaks for t155S and 300S. (**c**) Corresponding slope values of $I\text{p}/v^{1/2}$ for 155S and 300S at the A1 and C2 peaks

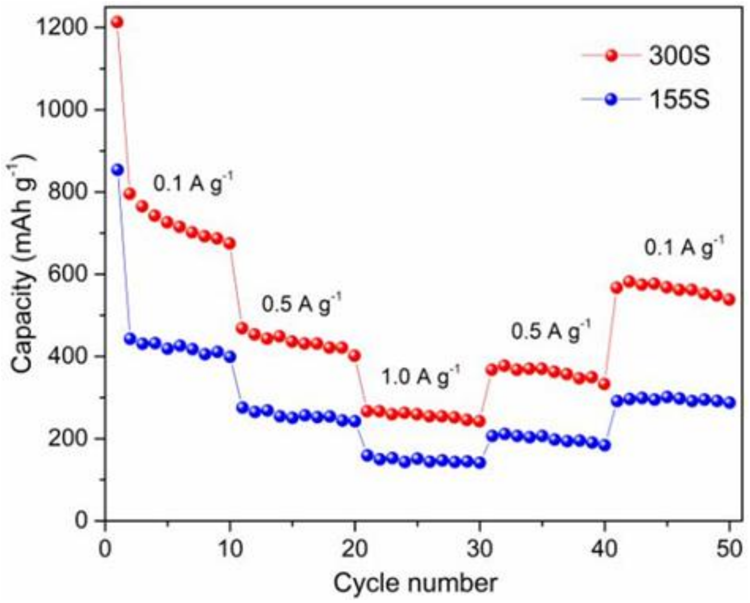

Fig. S6 Rate performances of the 155S and 300S electrodes in carbonate ester electrolytes

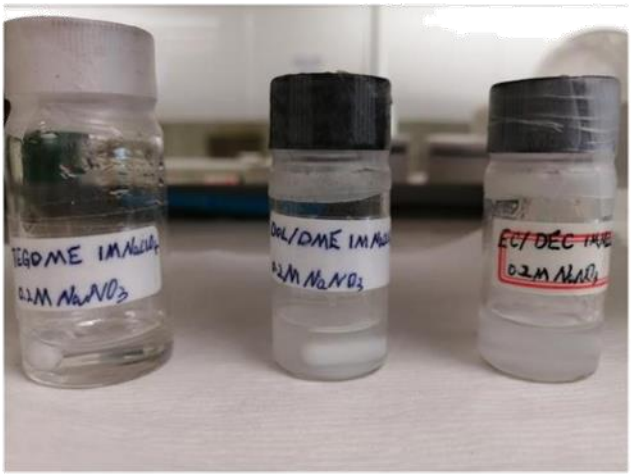

Fig. S7 Image of $NaNO_3$ dispersed in TEGDME, DOL/DME, and EC/DEC electrolytes with 1 M $NaClO_4$. $NaNO_3$ could only dissolve in TEGDME

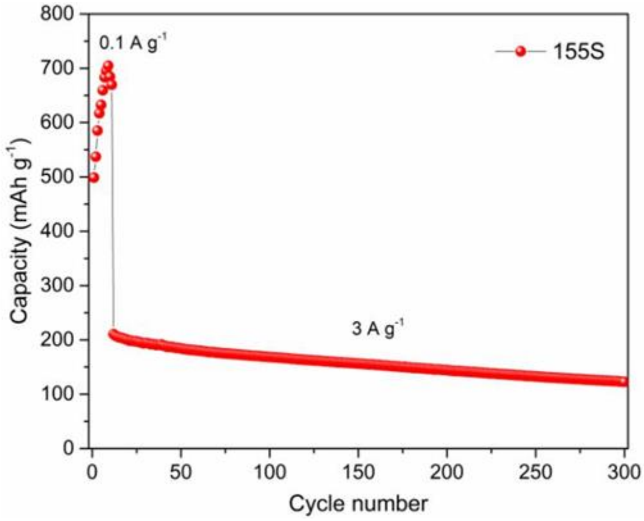

Fig. S8 Cycle performance of the 155S electrode in TEGDME electrolyte with $NaNO_3$ additive at the current density of 3.0 A g^{-1}

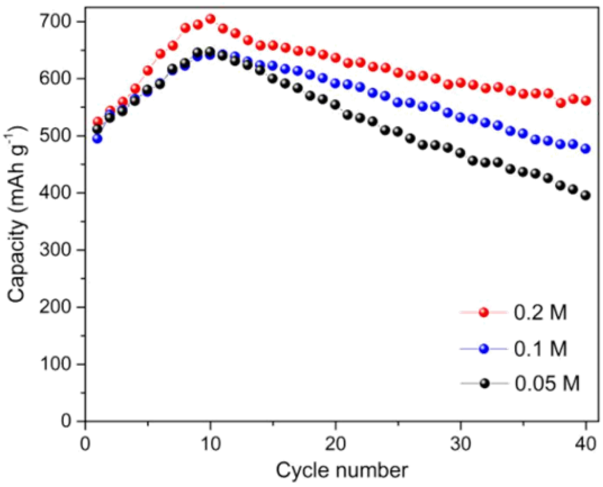

Fig. S9 Cycle performance of the 155S electrode in TEGDME electrolyte with 0.05, 0.1 and 0.2 M $NaNO_3$ additive, at the current density of 0.1 A g^{-1}

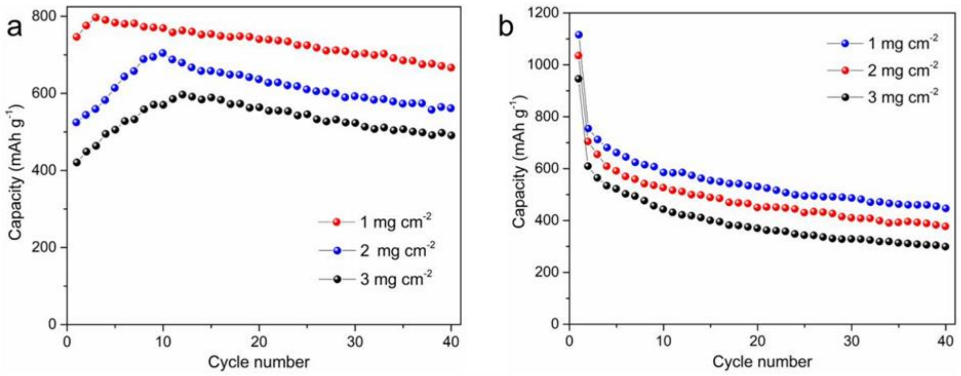

Fig. S10 Cycle performance of (**a**) the 155S electrodes and (**b**) the 300S electrodes with S content of 1 mg cm^{-2}, 2 mg cm^{-2} and 3 mg cm^{-2} in TEGDME with $NaNO_3$ additive

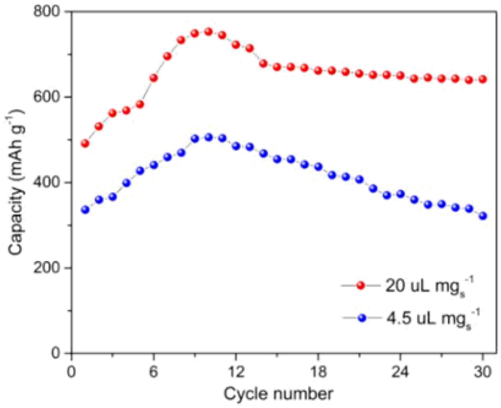

Fig. S11 Cycle performance of the 155S electrodes with 2 mg cm^{-2} sulfur, in 20 μL mgs^{-1} and 4.5 μL mgs^{-1} TEGDME electrolyte at 0.1 A g^{-1}, respectively

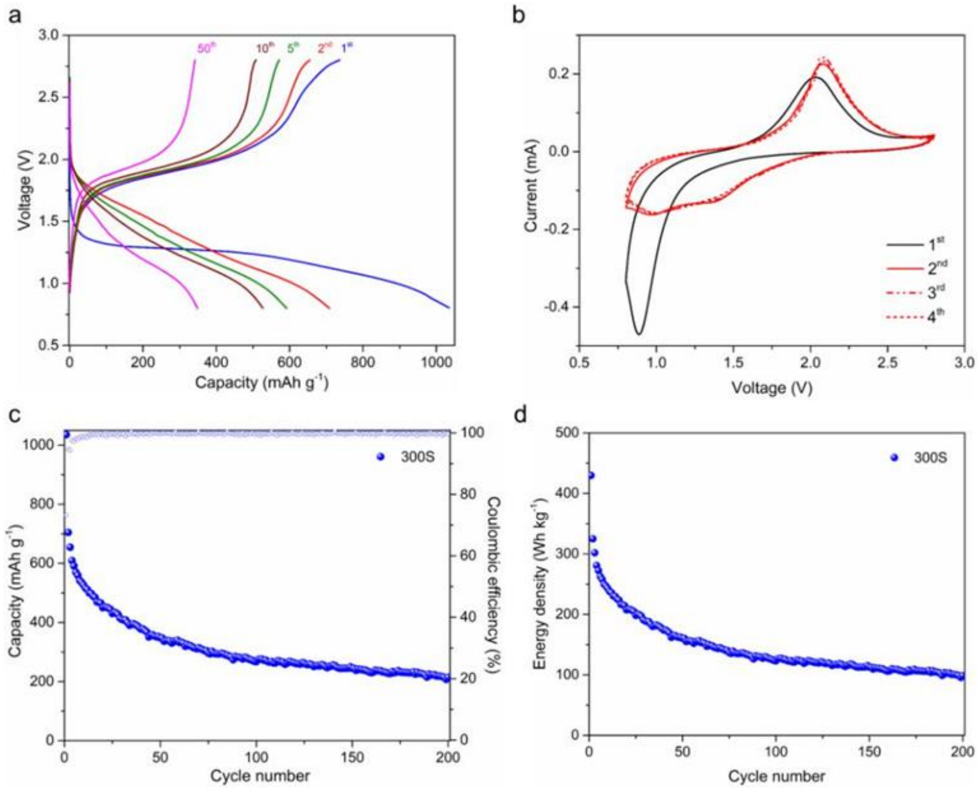

Fig. S12 (**a**) Voltage-capacity profiles and (**b**) CV profiles of the 300S electrode in TEGDME with 1 M $NaClO_4$ and 0.2 M $NaNO_3$ additive. (**c**) Cycling performance and (**d**) energy density of the 300S electrode in TEGDME with 1 M NaClO4 and 0.2 M $NaNO_3$ additive

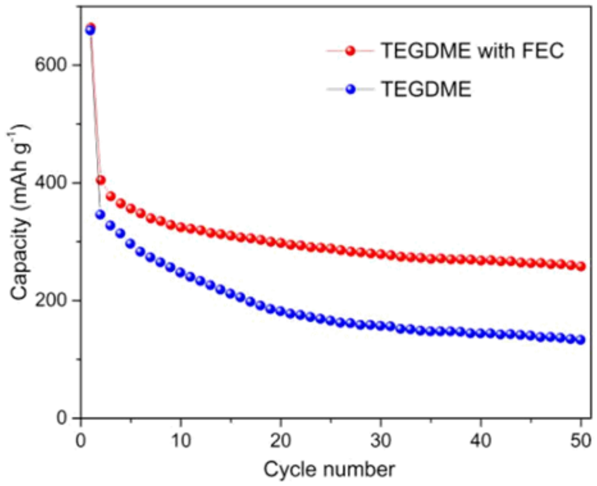

Fig. S13 Cycle performance of the 300S electrode in 1.0 M $NaClO_4$ and 0.2 M $NaNO_3$ TEGDME electrolyte with/without 5 wt % FEC additive at the current density of 1.0 A g^{-1}

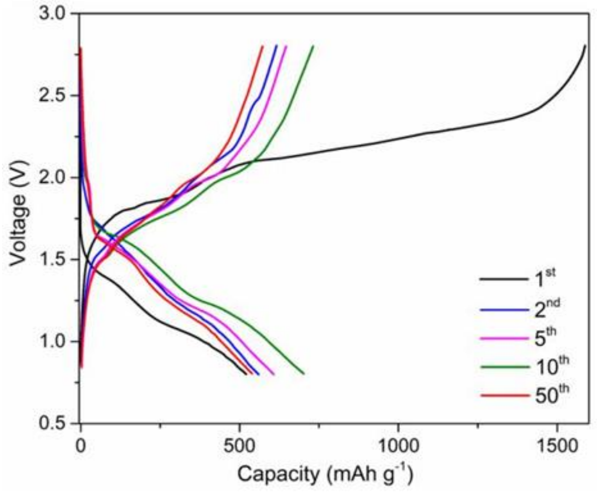

Fig. S14 Voltage-capacity profiles for the 155S electrode in TEGDME electrolyte without $NaNO_3$ additive at current density of 0.1 A g^{-1}

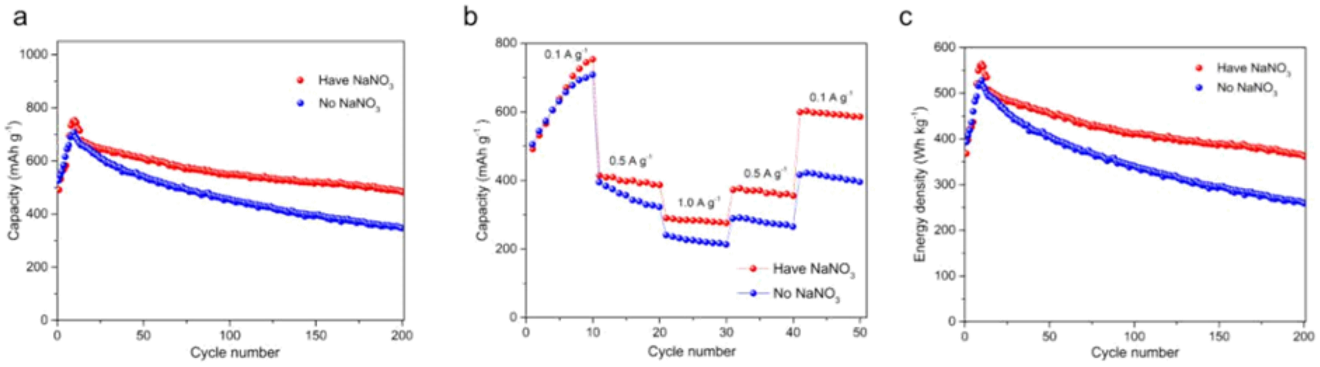

Fig. S15 (**a**) Cycle performance and (**b**) rate performance of the 155S in TEGDME with or without $NaNO_3$ additive based on the mass of sulfur. (**c**) Energy density of the 155S at 0.1 A g^{-1} based on the total mass of the electrode (including carbon black and binder)
